# Cell cycle-dependent localization of CHK2 at centrosomes during mitosis

**DOI:** 10.1186/1747-1028-8-7

**Published:** 2013-05-16

**Authors:** Guillaume Chouinard, Isabelle Clément, Julie Lafontaine, Francis Rodier, Estelle Schmitt

**Affiliations:** 1 Centre de recherche, Centre hospitalier de l'Université de Montréal (CRCHUM), Hôpital Notre-Dame et Institut du cancer de Montréal, Montréal, Québec, Canada; 2 Département de médecine, Université de Montréal, Montréal, Québec, Canada; 3 Département de radiologie, radio-oncologie et médecine nucléaire, Université de Montréal, Montréal, Québec, Canada

**Keywords:** CHK2, CHK1, Centrosome, Mitosis, Cell cycle

## Abstract

**Background:**

Centrosomes function primarily as microtubule-organizing centres and play a crucial role during mitosis by organizing the bipolar spindle. In addition to this function, centrosomes act as reaction centers where numerous key regulators meet to control cell cycle progression. One of these factors involved in genome stability, the checkpoint kinase CHK2, was shown to localize at centrosomes throughout the cell cycle.

**Results:**

Here, we show that CHK2 only localizes to centrosomes during mitosis. Using wild-type and CHK2^−/−^ HCT116 human colon cancer cells and human osteosarcoma U2OS cells depleted for CHK2 with small hairpin RNAs we show that several CHK2 antibodies are non-specific and cross-react with an unknown centrosomal protein(s) by immunofluorescence. To characterize the localization of CHK2, we generated cells expressing inducible GFP-CHK2 and Flag-CHK2 fusion proteins. We show that CHK2 localizes to the nucleus in interphase cells but that a fraction of CHK2 associates with the centrosomes in a Polo-like kinase 1-dependent manner during mitosis, from early mitotic stages until cytokinesis.

**Conclusion:**

Our findings demonstrate that a subpopulation of CHK2 localizes at the centrosomes in mitotic cells but not in interphase. These results are consistent with previous reports supporting a role for CHK2 in the bipolar spindle formation and the timely progression of mitosis.

## Background

The serine/threonine checkpoint kinases CHK1 and CHK2 are core components of the cell response to genotoxic stresses [1-3]. In response to DNA damage, CHK1 and CHK2 are activated by Rad3-related (ATR) and ataxia-telangiectasia mutated (ATM) protein kinases respectively, targeting many downstream substrates that coordinate cell cycle checkpoint activation, DNA repair and apoptosis [4,5]. Interestingly, recent studies have highlighted roles for CHK1 and CHK2 during normal cell cycle regulation in the absence of DNA damage. CHK1 has been reported to play a role in unperturbed S phase and in delaying the entry of cells into mitosis [6-10]. CHK1 has also been shown to be required for mitosis progression and for spindle assembly checkpoint function [11-14]. Recently, a role for CHK2 in the assembly of the bipolar mitotic spindle and normal mitosis progression has been reported [15]. This DNA-damage-independent function of CHK2 is mediated by the phosphorylation of the tumor suppressor BRCA1 on Ser 988, and is required to maintain chromosomal stability in the HCT116 human colorectal tumor cell line [15].

Centrosomes function primarily as microtubule-organizing centres and play a crucial role in the formation of bipolar spindles and chromosomal segregation during mitosis [16,17]. In addition, increasing evidence suggests that centrosomes also play roles in regulating various cell signalling pathways including cell cycle regulation and the DNA damage response [17-19]. Numerous cell cycle regulatory molecules have been identified at the centrosomes which are though to function as integration sites of positive and negative pathways to regulate cell cycle progression [17,18,20-25]. A growing number of components of the DNA damage response network, including p53, ATM, ATR, CHK1, CHK2, Rad 51, BRCA1 and BRCA2 have also been localized at the centrosomes supporting a role for centrosomes in the DNA damage response [8,9,26-35]. At the G2/M boundary, the molecular events that initiate entry into mitosis occur at the centrosomes where the activation of the mitotic kinase CDK1-cyclinB is initiated [36-39]. Interestingly, several studies report that a subpopulation of CHK1 localizes to the interphase centrosomes to regulate the entry of cell into mitosis by inhibiting centrosomal CDK1-cycline B activation [8-10].

Although CHK2 is mainly nuclear in somatic cells, several reports document the presence of a subpopulation of CHK2 at centrosomes. Strikingly, in embryonic stem (ES) cells, CHK2 has been shown to localize exclusively at the centrosomes and studies performed in *Drosophila melanogaster* embryos suggest that DmCHK2 localizes at the centrosomes to disrupt spindle assembly and chromosome segregation in response to DNA damage [29,31,40]. Using antibodies against CHK2-phospho-Thr68 in immunofluorescence experiments (IF), Tsvetkov and colleagues reported that a subpopulation of CHK2 localizes at centrosomes in interphase and mitotic cells in the absence of induced DNA damage [30]. In the same study, a centrosomal localization for a subset of HA-tagged CHK2 protein under specific IF conditions with pre-extraction of cells was reported [30]. The Thr383/387-phosphorylated form of CHK2 has also been localized at centrosomes in response to DNA damage, and CHK2 was found to co-purify with centrosomes in gradient-purified centrosome preparations [8,41]. Despite these observations, the centrosomal localization of CHK2 remains controversial and there are doubts regarding the specificity of the antibodies used to demonstrate expression of CHK2 at the centrosomes by immunofluorescence. In the present study we show that anti-CHK2 antibodies used to stain CHK2 at centrosomes cross-react non-specifically with an unknown centrosomal protein(s). By using cells lines expressing Flag or GFP-tagged CHK2 proteins, we demonstrate that CHK2 localizes exclusively in the nucleus of cells in interphase. However, we observed that a small portion of CHK2 localizes to centrosomes in mitotic cells, from late prophase until cytokinesis supporting a role for CHK2 during mitosis [15].

## Results

### CHK2 is localized in the nucleus but not at the centrosomes in interphase cells

**Figure 1:**
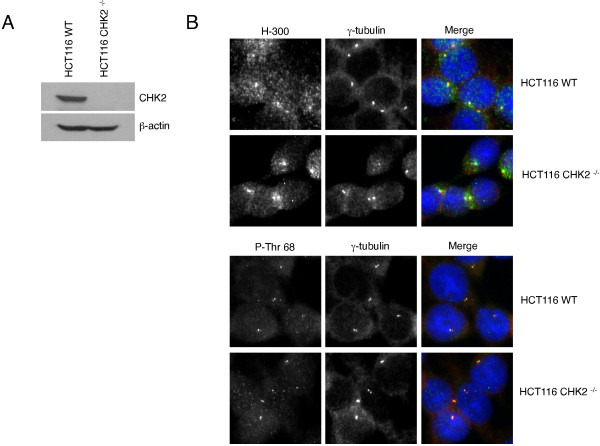
**Centrosomes stain positive for CHK2 in human colon cancer cells deficient in CHK2.** (**A**) There is no expression of CHK2 in HCT116 CHK2^−/−^ cells. HCT116 WT and HCT116 CHK2 knockout cells were subjected to Western blotting with anti-CHK2 antibody. (**B**) We did not detect differences in the staining between WT and CHK2 knockout cells. Exponentially growing HCT116 WT and CHK2^−/−^ cells were fixed and immunostained with antibodies against the N-terminal portion of CHK2 (H-300) or the Thr68-phosphorylated form (P-Thr 68) (green). Cells were costained for γ-tubulin (red) and counterstained with DAPI (blue).

**Figure 2:**
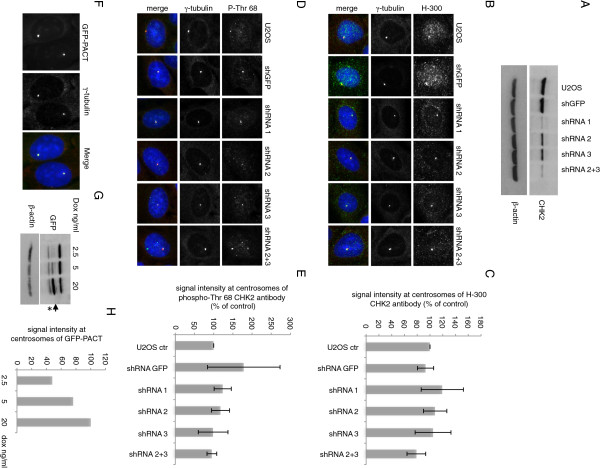
**Centrosomal staining for CHK2 antibodies persists in cells expressing CHK2 targeting shRNAs.** U2OS cells were stably transduced with various shRNAs directed against CHK2 or GFP. (**A**) Western blots showing the expression level of endogenous CHK2 in the transduced cell lines. (**B** and **D**) Cells were fixed and immunostained with the indicated anti-CHK2 antibodies (green) and costained for centrosomes (red) and DNA (blue). Representative fields of the immunofluorescence stainings obtained with the indicated cell lines are shown. (**C** and **E**) The fluorescence intensity of the CHK2 antibodies H-300 and P-Thr 68 was quantified and represented as a ratio of the control γ-tubulin signal at centrosomes. This ratio was set at 100% in control untransduced cells. Results are representative of 3 independent experiments where the intensity at the centrosomes was monitored in 200 cells. Error bars represent the standard deviation from the mean of 3 experiments. To validate the method of quantification of the fluorescence intensity signal at centrosomes, stable transduced GFP-PACT U2OS cells were generated. (**F**) A representative field showing the centrosomal localization of the GFP-PACT fusion protein. 48 h after 20 ng/ml doxycycline addition, cells were fixed and stained with anti-γ-tubulin antibody (red) and DAPI (blue). GFP-PACT was visualized by direct fluorescence. (**G**, **H**) GFP-PACT expression was induced with 2.5, 5 or 20 ng/ml doxycycline and cells were stained as in (**F**). (**G**) The expression of GFP-PACT at each doxycycline concentration was analyzed by Western blotting using anti-GFP antibody. The arrow indicates the bands corresponding to GFP-PACT and the asterisk designates non-specific signal. (**H**) The fluorescence intensity at centrosomes of GFP-PACT was quantified as in C and E and set at 100% in cells induced with 20 ng/ml doxycycline. Graphs represent the mean of intensity ± s.d. of 3 independent experiments.

Several antibodies raised against CHK2 have been reported to stain the centrosomes by immunofluorescence in HEK293T and U2OS cells [8,30]. We confirm that both the rabbit polyclonal affinity-purified antibodies raised against the N-terminal amino acids 1 to 300 of CHK2 (H-300) as well as the CHK2-phospho-Thr68 antibody stain the centrosomes of U2OS cells fixed with 100% methanol. To validate the specificity of these antibodies in immunofluorescence experiments, we used an isogenic human colorectal cancer HCT116 cell line with a targeted deletion of CHK2 [42]. The expected 62 kDa band corresponding to the molecular weight of CHK2 protein was not detected in the HCT116 CHK2^−/−^ cells by Western blotting, but was detected in the wild type (WT) cells (Figure 1A). Although we obtained positive staining at the centrosomes with the CHK2 H-300 and phospho-Thr68 antibodies in IF experiments there was no significant difference in the staining at the centrosomes between the HCT116 WT and CHK2 knockout cells (Figure 1B). To confirm the results obtained with the HCT116 cell lines, we generated human osteosarcoma (U2OS) cell lines stably transduced with small hairpin *CHK2* RNAs targeting different regions of *CHK2* ORF. Lentiviral shRNAs directed against N-terminal sequences of the *CHK2* ORF located either at 3′ (shRNA-1) or 5′ (shRNA-2) of Thr68 were used to infect U2OS cells. U2OS cells lines transduced with a CHK2 shRNA targeting the 3′UTR sequence (shRNA-3) or with a combination of shRNA 2 + 3 were also generated. We confirmed the shRNA-mediated silencing of CHK2 in the stably transduced cells by immunoblotting, and noted that the efficiency of CHK2 depletion varied between small hairpins with shRNA-1 providing the best depletion (Figure 2A). We then stained the cell lines generated with the anti-CHK2 H-300 and phospho-Thr68 antibodies. Although the intensity of staining with the H-300 antibody in the U2OS cell lines depleted for CHK2 decreased in the nucleus, the centrosomes remained positively stained in all cell lines. There was no significant decrease of the centrosomal signal in CHK2-depleted cell lines compared to the untransduced U2OS cells or cells transduced with control GFP-shRNAs (Figure 2B-E). To validate the methodology we used to quantify the fluorescence intensity signal at the centrosomes, we generated a stable U2OS cell population expressing a doxycycline-inducible GFP protein fused to the PACT (pericentrin-AKAP450-centrosome targeting) domain of AKAP450 [43]. Confirming previous reports, we found that the GFP-PACT fusion protein localized predominantly at the centrosomes (Figure 2F). We induced the expression of the GFP-PACT fusion protein by adding increasing doses of doxycycline to the culture media. We observed a dose-dependent increase in the expression of the GFP-PACT fusion protein that was proportional to the intensity of the fluorescence measured at the centrosomes (Figure 2G and H). In summary, the staining pattern we obtained with the U2OS cell lines is in agreement with that observed in the HCT116 cells. We confirm that the CHK2 H-300 and CHK2-phospho-Thr68 antibodies bind non-specifically to protein(s) other than CHK2 localized at the centrosomes in interphase cells.

### CHK2 localizes at the centrosomes in mitotic cells

**Figure 3:**
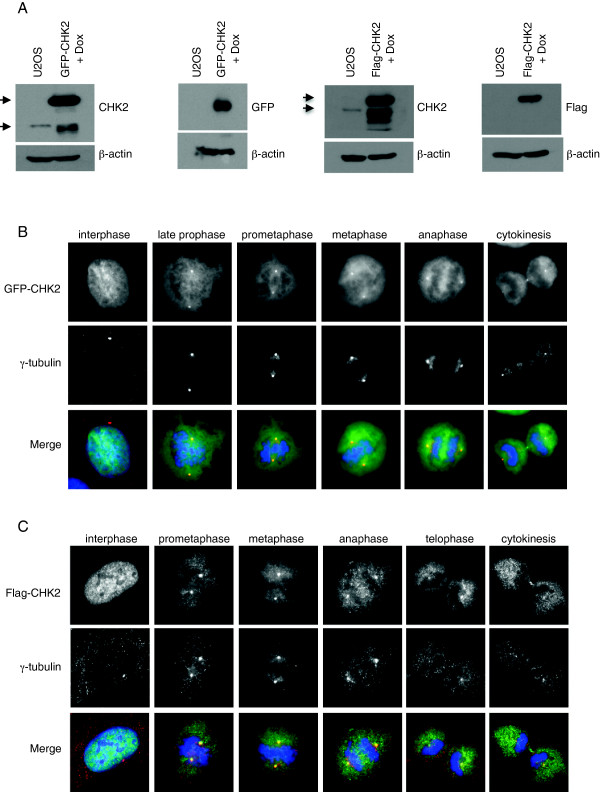
**CHK2 localizes at centrosomes in mitotic cells but not in interphase.** U2OS cells were transduced with lentiviruses coding for GFP-CHK2 or Flag-CHK2 and stably selected. (**A**) Cells were exposed for 48 h to doxycycline at 5 ng/ml for GFP-CHK2 cells, or 20 ng/ml for Flag-CHK2 cells, and expression of proteins in whole cell extracts was analyzed by Western blotting using the indicated antibodies. The arrows indicate endogenous and exogenous CHK2 proteins. Note that some exogenous CHK2 is expressed without tag. β-actin was used as loading control. (**B** and **C**) GFP-CHK2 and Flag-CHK2 are exclusively localized in the nucleus of interphase cells whereas a small subpopulation localizes at the centrosomes during mitosis. 48 h following transgene induction cells were fixed in methanol. Centrosomes were stained with anti-γ-tubulin antibody (red), and DNA was counterstained with DAPI (blue). The localization of GFP-CHK2 was observed by direct fluorescence and Flag-CHK2 was immunostained with an anti-Flag antibody (green). Cells in interphase and various phases of mitosis were selected.

To examine more closely the localization of CHK2, we generated stable U2OS cell lines that express the fusion protein GFP-CHK2 or Flag-CHK2 under the control of the doxycyclin-inducible promoter (Figure 3A). We first assessed the functionality of the GFP and Flag-tagged CHK2 proteins by examining their phosphorylation status in response to DNA damage induced by ionizing radiation (γ-IR). In response to gamma rays, the exogenous CHK2 fusion proteins were found phosphorylated on Thr 68, Thr 383/387 and Ser 516 suggesting that the kinases are fully active (see Additional file 1). In addition, we detected an increase in the phosphorylation of the CHK2 substrate CDC25 A (Ser 123) indicative of GFP- and Flag-CHK2 activation in the transduced cell lines relative to U2OS control cells (see Additional file 1). These data suggest that the doxycycline-inductible GFP-CHK2 and Flag-CHK2 proteins are functional kinases [2,3].

**Figure 4:**
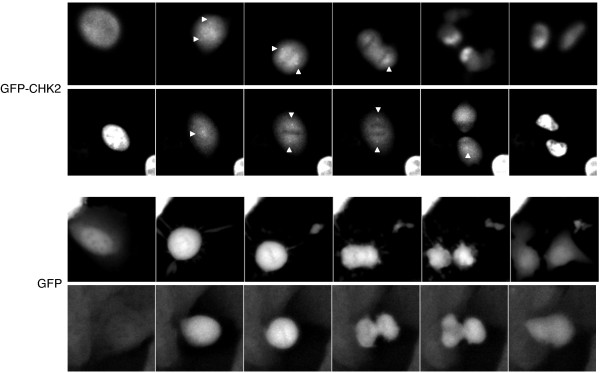
**GFP-CHK2 localizes at centrosomes and spindle in mitotic living cells.** Representative images of the live cell videos showing mitotic U2OS cells expressing GFP-CHK2 or GFP proteins. Arrowheads indicate centrosomes.

In direct immunofluorescence experiments performed 48 h following doxycycline addition to the culture medium, we found GFP-CHK2 to be exclusively localized in the nucleus of interphase cells with no apparent centrosomal staining (Figure 3B). However, we detected GFP-CHK2 at the centrosomes in mitotic cells. We found a small subpopulation of GFP-CHK2 at the centrosomes from the late prophase/early prometaphase stage, where centrosomes separate and migrate to cell poles, until cytokinesis. We also observed GFP-CHK2 concentrated at the mid-body in late mitosis (Figure 3B). Similar results were obtained in immunostained cells expressing Flag-CHK2 fusion protein (Figure 3C). The centrosomal localization of GFP-CHK2 and Flag-CHK2 in mitotic cells was maintained when microtubules were depolymerized by nocodazole treatment, suggesting that CHK2 localizes to mitotic centrosomes in a microtubule-independent manner (see Additional file 2). To further control the reliability of these results, we also generated U2OS cell lines transduced with the doxycycline-inductible GFP, GFP-CHK1 and Flag-CHK1 constructs. Unlike GFP-CHK2 and Flag-CHK2, the GFP, GFP-CHK1 and Flag-CHK1 proteins, although induced at high levels in the U2OS cell lines, did not localize to the centrosomes in mitotic cells (see Additional file 3). In interphase cells expressing very high levels of GFP protein, some centrosomes appeared slightly stained but most cells did not stain at the centrosomes (see Additional file 3). Although these results suggest that the centrosomal localization revealed for GFP-CHK2 and Flag-CHK2 is specific, we decided to further support our findings by performing live-cell microscopy experiments. In living cells, we observed that a fraction of GFP-CHK2 was associated with the centrosomes during mitosis from early prometaphase until anaphase. In metaphase, GFP-CHK2 also localizes at the bipolar mitotic spindle. In contrast to GFP-CHK2, no specific enrichment of GFP at any particular structure was detected in mitotic cells (Figure 4 and Additional movie files 4 and 5). Collectively, our results indicate that a subpopulation of CHK2 localizes at centrosomes specifically in mitotic cells. These data are consistent with the novel function proposed for CHK2 in the mitotic spindle assembly and the progression of mitosis reported recently by others [15].

### Polo kinase 1 is required for CHK2 localization at centrosomes

**Figure 5:**
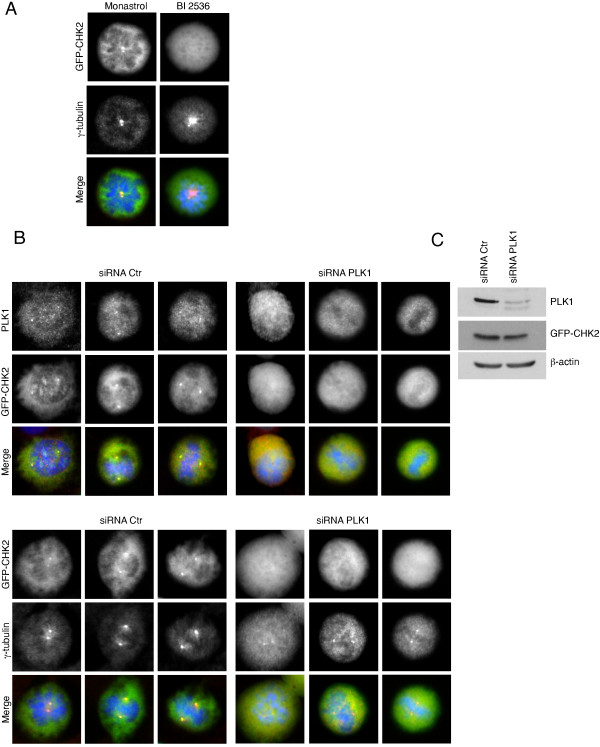
**Polo kinase 1 is required for CHK2 localization at centrosomes.** (**A**) Inhibition of PLK1 activity with BI 2536 inhibits GFP-CHK2 association with centrosomes. U2OS GFP-CHK2 cells were incubated with doxycycline at 5 ng/ml for 32 h and treated for an additional 16 h with BI 2536 (100 nM) or monastrol (100 μM) in doxycycline-containing medium. Cells were then fixed and stained for centrosomes (red) and DNA (blue) and GFP-CHK2 was visualized by direct fluorescence (green). (**B**) Inhibition of PLK1 expression prevents GFP-CHK2 association with centrosomes during mitosis. U2OS GFP-CHK2 cells were transfected with a siRNA directed against *PLK1* or control siRNA. 48 h post-transfection cells were fixed to examine GFP-CHK2 localization by fluorescence microscopy as in (**A**). (**C**) Western blot analysis of PLK1 expression shows a significant decrease in the expression of PLK1. Cell lysate from PLK1 siRNA-transfected U2OS cells was prepared from arrested mitotic cells collected by shake-off 48 h post-transfection. As a control, U2OS cells transfected with control siRNA were synchronized in mitosis by a 16 hours nocodazole block and protein extracts were prepared from the mitotic cells collected by shake-off.

Polo-like kinase 1 (PLK1) is a key mitotic kinase that regulates mitotic entry and mitosis progression. PLK1 localizes to centrosomes and kinetochores in early mitotic stages and at the central spindle and mid-body in anaphase and cytokinesis [44,45]. Since PLK1 localizes at the centrosomes and regulates CDK1-Cyclin B activation, centrosome maturation and separation as well as spindle assembly, we examined whether it is required for the recruitment of CHK2 at mitotic centrosomes. U2OS cells induced for GFP-CHK2 expression were treated with BI 2536 an inhibitor of PLK1, or monastrol an inhibitor of the Eg5 kinesin [46-48]. We found that both treatments inhibit centrosomes separation resulting in the formation of monopolar spindles and the arrest of cells in prometaphase (Figure 5A). In cells treated with monastrol, GFP-CHK2 was present at the unseparated centrosomes of the monopoles while it was not associated with centrosomes in BI 2536-treated cells, suggesting that PLK1 kinase activity is required for CHK2 recruitment at the centrosome during mitosis. To confirm the role of PLK1 in targeting CHK2 at the centrosome, we depleted the expression of PLK1 using short interfering RNA (siRNA).

Unlike BI 2536 that strongly inhibits centrosome separation, cells transfected with siRNA against *PLK1* arrested at late prophase/early prometaphase with unseparated centrosomes or at metaphase showing effective centrosome separation (Figure 5B and Additional file 6). Accumulation of these cells in metaphase most likely results from the incomplete silencing of PLK1 by the siRNA, as observed on the Western blots (Figure 5C and Additional file 6B). Alternatively, a potential off-target effect of BI 2536 is also possible [46]. In addition, PLK1 silencing or inhibition has no effect on centrosomes in interphase (Additional file 7). Most importantly, in all mitotic-arrested cells following siRNA-mediated PLK1 depletion or inhibition, the association of GFP-CHK2 with centrosomes was inhibited suggesting that PLK1 activity is required for CHK2 recruitment at the centrosomes during mitosis. However, data presented in Additional files 6 and 7 indicate that CHK2 silencing does not influence centrosome duplication and separation.

## Discussion

Increasing evidence indicates that centrosomes integrate pathways that regulate cell cycle progression in interphase and mitosis. In the past years, key proteins of the DNA damage response have been localized at the centrosomes supporting a role for DNA damage responsive molecules in the control of unperturbed cell cycle progression and mitosis. Several studies revealed a centrosomal localization for CHK2 in interphase and mitotic cells [30,31,41]. We show here that the CHK2 rabbit polyclonal antibodies H-300 and CHK2-phospho-Thr68 stain the centrosomes by cross-reacting with an unknown centrosomal protein(s). By using U2OS cells transfected with Flag and GFP-tagged CHK2 fusion proteins we demonstrate that a subpopulation of CHK2 localizes at the centrosomes specifically in mitotic cells, from the late prophase/early prometaphase stage until cytokinesis. Moreover, we show that the centrosomal localization of CHK2 depends on PLK1 activity.

Our finding is in agreement with a recent work published by Stolz and al., supporting a new function for CHK2 in the mitotic bipolar spindle assembly and the accurate segregation of chromosomes in the human colon cancer cell line HCT116 [15]. In this study, the tumor suppressor BRCA1 has been identified as a mitotic target of CHK2, and alteration of the CHK2-BRCA1 pathway was shown to promote chromosomes segregation errors in dividing cells, a feature that is commonly observed in cancer cells and might drive chromosomal instability and cellular transformation [15,49,50]. Whether this new function of CHK2 in mitosis is associated with the centrosomal pool of CHK2 remains to be established. Interestingly, the CHK2 substrate BRCA1 has also been localized at the centrosomes in mitotic cells where it was reported to inhibit microtubule nucleation, an event that initiates entry into mitosis [26,51-54].

The kinase CHK2 is a checkpoint kinase known for its functions in the DNA damage response and is detected in the nucleus of interphase cells. In response to DNA damage CHK2 phosphorylates several targets *in vivo* including the phosphatases CDC25 A and C, the tumor suppressor BRCA1, the promyelocytic leukemia protein (PML), as well as the transcription factors p53, E2F1 and FOXM1 which are all involved in cell cycle checkpoints, programmed cell death and DNA repair [55-60]. More recently, CHK2 has been demonstrated to be required for the proper progression of mitosis in HCT116 cells [15]. Interestingly, recent findings indicate that several other key proteins of the DNA damage response, including CHK1, ATM, DNA-PK, MDC1 and BRCA2 also play roles in regulating the proper progression of mitosis and/or the mitotic spindle assembly checkpoint (SAC), supporting the existence of a functional crosstalk between proteins of the DNA damage response and the mitotic regulatory network [8-13,22,61-66].

## Conclusion

Despite several recent reports documenting the presence of CHK2 at centrosomes there was some doubt about the specificity of the antibodies that stained positively for CHK2 at the centrosomes by immunofluorescence. In this study we conclusively show that CHK2 does not localize at the centrosomes in interphase cells, but rather, that a sub-population of CHK2 associates with centrosomes in a PLK1-dependent manner during mitosis.

## Methods

### Cells culture and reagents

Human osteosarcoma U2OS cells used in all experiments stably express the tetracycline (Tet) repressor. U2OS T-REx cells were obtained from Dr. Eric Campeau (University of Massachusetts, USA). HCT116 WT and CHK2^−/−^ cells were a gift from Dr. Fred Bunz (Johns Hopkins University, Baltimore, USA) to Dr. Francis Rodier. Cells were maintained at 37°C, 5% CO_2_ in Dulbecco’s modified Eagle’s Medium supplemented with 10% fetal calf serum. BI 2536 (Axon Medchem) and monastrol (Enzo Life sciences) were used at 100 nM and 100 μM respectively. Nocodazole was purchased from Sigma-Aldrich.

### Generation of U2OS cell lines

To generate inducible U2OS cells lines expressing CHK2 or CHK1 fused to the GFP or FLAG tag, CHK1 and CHK2 cDNAs were cloned into the entry vectors pENTR4-GFP-C3 (Addgene # w393-1) and pENTR4-FLAG (Addgene # w210-2) respectively and the entry vectors were recombined into the inducible destination vector pLenti CMV/TO Puro (Addgene # 670–1). To generate inducible U2OS cells expressing GFP-PACT fusion protein, the PACT domain (amino acids 3643–3808) of AKAP450 was amplified from the GFP-AKAP450 vector provided by Dr. S. Munro (Cambridge, United Kingdom) with the following primers: forward, 5′-AAGCTTGCCAACATTGAAGCCATCATTGCC-3′ and reverse, 5′-GAATTCTTATGCACCTTGATTCAGTCCAAAGC-3′. The amplified sequence was cloned into the entry vector pENTR4-GFP-C3 (Addgene # w393-1) that was recombined into the destination vector pLenti CMV/TO Puro (Addgene # 670–1). Lentiviruses for all the constructs were generated with virus titers to infect 50 to 80% of cells and stably transduced cells were selected with 0.5 microgram /ml puromycin [67].

### RNA interference

RNAi lentiviruses previously shown to effectively reduce CHK2 levels were purchased from Open Biosystems [68]. The short hairpin target sequences used were:

shRNA-1 # TRCN0000039946: 5′-GCCAATCTTGAATGTGTGAAT-3′ ;

shRNA-2 #TRCN0000010213: 5′-ACGATGCCAAACTCCAGCCAG-3′ ;

shRNA-3 #TRCN0000010314: 5′-ACTCCGTGGTTTGAACACGAA-3′.

For PLK1 knockdown, cells were transfected with a siRNA targeting the sequence 5′-AGAUUGUGCCUAAGUCUCU-3′ [69,70]. Transfection of siRNAs was carried out using DharmaFECT reagent (Thermo Scientific, Dharmacon) according to manufacturer’s instructions.

### Immunofluorescence microscopy

Cells were grown on high precision 18 mm/1.5H glass coverslips (Marienfeld) and fixed in −20°C methanol for 15 min and blocked with PBS containing 3% BSA, 2% FCS, 0.3% Triton X-100. Fixed cells were incubated for 1 hour at room temperature with the indicated primary antibodies diluted in blocking buffer, and for 45 min with the Alexa Fluor-conjugated secondary antibodies. DNA was stained for 5 min with DAPI at 100 ng/ml (Sigma). Images were acquired using a Nikon Eclipse 600 fluorescence microscope equipped with a CoolSNAP HQ^2^ camera (Photometrics) and processed using NIS-Element AR 3.0 and Photoshop (CS5, Adobe). Cells were immunostained using the following primary antibodies: anti-γ-tubulin (GTU-88, Sigma T6557, 1:2000), anti-CHK2 (H300, Santa Cruz, 1:50), anti-phospho-Thr68-CHK2 (Cell signaling 1:50), anti-α-tubulin (DM1A, Sigma 1:500), anti-Flag (M2, Sigma, 1:1000), anti-Flag (F7425, Sigma, 1:250), anti-PLK1 (#06-813, Millipore 1:100) and the secondary antibodies Alexa Fluor 488/TxRed (Molecular Probes, 1: 800). For the quantification of fluorescence signal intensities at the centrosomes, unsaturated images were acquired with the same exposure settings. The areas corresponding to the centrosomes were defined with the γ-tubulin stainings and fluorescence intensities within the determined centrosomal areas were measured using NIS-Element software.

### Western blotting

To prepare protein extracts, cells were lysed in lysis buffer containing 50 mM Tris–HCl pH 7.4, 150 mM NaCl, 1% Triton X-100, 2.5 mM Na_3_VO_4_, 10 mM NaF, 5 mM sodium pyrophosphate, 2 mM phenylmethylsulfonyl fluoride (PMSF), 10 μM MG132, 20 mM β-glycerophosphate, and a cocktail of proteases inhibitors (Complete™ Roche Applied Science). Proteins were run on 10% SDS-PAGE gels, transferred on nitrocellulose membranes and analyzed by immunoblotting. The antibodies used were anti-GFP (ab290, abcam, 1:1000), anti-Flag (M2, Sigma, 1:1000), anti-CHK2 (B-4, Santa Cruz, 1:500), anti-CHK1 (DCS-310, SantaCruz 1:500), anti-phospho-Thr68-CHK2 (Cell signalling, 1:1000), anti-phospho-Thr387-CHK2 (Assay bioTech, 1:500), anti-phospho-Ser516-CHK2 (Cell signalling, 1:1000), anti-phospho-Ser123-CDC25A (MJS Biolynx 1:250), anti-PLK1 (#06-813, Millipore, 1:500) and anti-β-actin (AC-74, Sigma, 1:20 000).

### Time-lapse microscopy

For live cell videomicroscopy, U2OS cells transduced with GFP-CHK2WT or control GFP were grown on CellView™ 35 mm glass bottom dishes (170 ± 10 μM bottom thickness) from Greiner-Bio One. Protein expression was induced by a 48 h doxycycline treatment and cells were synchronized at the G1/S transition using a single 24 h thymidine block. 8 h after release from thymidine block they were incubated in FCS-supplemented, phenol red-free Dulbecco’s modified Eagle’s Medium and placed at 37°C in a heated chamber maintained at 5% CO_2_. Images were collected on a Zeiss Axio Observer Z1 automated microscope every 2.5 minutes for 16 hours using a Plan-Apochromat 20x/0.8NA objective, a Zeiss HRm Axiocam and LED pulsed light illumination setup.

### Statistical analysis

Statistical analyses were performed using a two-tailed Student t-test, type 3. Difference with P < 0.05 is considered as significant.

## Abbreviations

IF: Immunofluorescence; ORF: Open reading frame; γ-IR: Gamma-irradiation; PLK1: Polo-like kinase 1

## Competing interests

The authors declare that they have no financial and non-financial competing interests.

## Authors’ contribitions

GC and IC carried out the experiments. GC developed the method used to quantify centrosomal fluorescence and participated to the design of the experiments. IC and JL established the transduced cell lines. FR generated lentiviruses expressing CHK2 shRNAs and was involved in revisiting the manuscript critically. ES conceived the study, designed the experiments and wrote the manuscript. All authors read and approved the final manuscript.

## Supplementary Material

Additional file 1 **The exogenous GFP-CHK2 and Flag-CHK2 fusion proteins are functional kinases.** U2OS GFP-CHK2 and U2OS Flag-CHK2 cell lines were incubated with doxycycline for 48 h to induce transgene expression and cells were exposed to γ-IR (10 Gy ). (**A**) 1 h after genotoxic insult whole-cell lysates were prepared and phosphorylation of CHK2 fusion proteins on Thr 68, Thr 383/387 and Ser 516 was assessed by Western blotting using the indicated antibodies. The arrows indicate GFP-CHK2 and Flag-CHK2 proteins phoshorylated on Thr383/387. (**B**) The phosphorylation of the CHK2 substrate CDC25 A (Ser123) was analyzed by Western blotting of protein extracts prepared 8 h after irradiation. The arrow indicates the band corresponding to P-Ser123-CDC25A. Both GFP-CHK2 and Flag-CHK2 retain full kinase activity. Click here for file

Additional file 2 **During mitosis the localization of CHK2 at the centrosomes is microtubules independent.** Doxycycline-induced U2OS GFP-CHK2 and U2OS Flag-CHK2 cell lines were incubated for 16 h in nocodazole (0.3 μM) to arrest cells in prometaphase. Cells were treated for an additional hour with 10 μM nocodazole prior to be fixed and stained with anti-γ-tubulin antibody (red) to stain the centrosomes. GFP-CHK2 was visualized by direct fluorescence and Flag-CHK2 was immunostained with an anti-Flag antibody (green). To control microtubules depolymerization cells were also stained for α-tubulin. Click here for file

Additional file 3 **GFP, GFP-CHK1 and Flag-CHK1 do not localize to the centrosomes.** U2OS stably transduced with lentiviruses coding for GFP, GFP-CHK1 or Flag-CHK1 were exposed to doxycycline at 5 ng/ml, 10 ng/ml and 20 ng/ml. (**A**) 48 h following doxycycline addition cells were collected. The expression of exogenous proteins was analyzed by Western blotting using the indicated antibodies. The arrows denote endogenous and exogenous CHK1 proteins. β-actin was used as loading control. (**B-D**) 48 h post-induction, cells were fixed and immunostained with anti-γ-tubulin antibody (red) and costained with DAPI (blue). The localization of GFP and GFP-CHK1 was observed by direct fluorescence and Flag-CHK1 was immunostained with an anti-Flag antibody (green). Cells in interphase and various phases of mitosis were selected. Click here for file

Additional file 4 **Time-lapse movie showing GFP-CHK2 at centrosomes in mitotic U2OS cells.** U2OS GFP-CHK2 were incubated with doxycycline for 48 h and synchronized by a single 24 h thymidine block. When the synchronized cell population progressed through late G2 phase and mitosis, images were acquired every 2 minutes with a Zeiss Axio Observer Z1 automated microscope. Click here for file

Additional file 5 **Time-lapse movie showing a mitotic U2OS cell expressing control GFP protein.** Cells were imaged in the same conditions as for Additional file 4. Click here for file

Additional file 6 **Quantification of centrosome separation in mitotic cells.** (**A**) Control U2OS cells or cells stably transduced with CHK2 shRNA 1 or CHK2 shRNA 2 + 3 were transfected with a siRNA directed against *PLK1* or incubated with BI 2536 (100 nM). 24 h following transfection or 16 h after treatment with BI 2536, cells were fixed and stained with anti-γ-tubulin antibody and DAPI. Representative images of the mitotic-arrested cells are shown. The percentage of each mitotic cellular population was measured. Error bars represent the mean ± s.d. of 3 independent experiments, each experiment monitoring 200 mitotic cells (*P < 0.05; ^_^ P > 0,05). (**B**) Western blot analysis of PLK1 expression. Cell lysates from PLK1 siRNA-transfected U2OS cells were prepared from mitotic cells collected by shake-off 24 h post-transfection. Protein extracts prepared from asynchronous cells or mitotic cells collected by shake-off 24 h following nocodazole treatment serves as control. Click here for file

Additional file 7 **Quantification of centrosomes duplication/separation in interphase.** (**A**) Experimental procedure. Control U2OS cells or cells stably transduced with CHK2 shRNA 1 were synchronized at the G1/S boundary by a double thymidine block (DTB). At the indicated times during the cell cycle synchronization protocol, cells were transfected with control or PLK1 siRNAs, incubated with BI 2536 or left untreated. (**B**) After release from second thymidine block, cell synchronization was confirmed by FACS analysis at the indicated times. (**C**) The inhibition of PLK1 expression was confirmed by Western blotting. Cell lysates from PLK1 siRNA-transfected cells were prepared from mitotic cells collected by shake-off 11,5 h after release from DTB. Protein extracts prepared from mitotic cells collected 24 h following nocodazole treatment serves as control. (**D**) At each time point after release, cells were fixed and stained with anti-γ-tubulin antibody and DAPI. The interphase cells with one or two unseparated/separated centrosomes were divided in 4 patterns, as shown in representative images, and cells in each pattern were quantified. Error bars represent the mean ± s.d. of 3 independent experiments, each experiment monitoring 200 interphase cells. Click here for file

## Ackowledgements

We thank Dr S. Munro (Cambridge, United Kingdom) for providing the vector coding for the PACT domain of AKAP450. We are also grateful to Eric Campeau (University of Massachusetts, USA) for the gift of the U2OS TREx cell line and to Fred Bunz (Johns Hopkins University, Baltimore, USA) for the HCT116WT and HCT116CHK2−/− cell lines. We thank Dr Richard Bertrand (CRCHUM) for advice and editorial work. This work was supported by the Canadian Institutes of Health research and the Institut du cancer de Montréal. ES received support as a research scholar of the FQRS. We also thank Mrs Suzana Anjos for her editorial work.

## References

[B1] Chen Y Poon RY The multiple checkpoint functions of CHK1 and CHK2 in maintenance of genome stability Front Biosci 2008 13 5016 5029 18508566 10.2741/3060

[B2] Bartek J Falck J Lukas J CHK2 kinase-a busy messenger Nat Rev Mol Cell Biol 2001 2 877 886 10.1038/35103059 11733767

[B3] Ahn J Urist M Prives C The Chk2 protein kinase DNA Repair (Amst) 2004 3 1039 1047 10.1016/j.dnarep.2004.03.033 15279791

[B4] Shiloh Y ATM and ATR: networking cellular responses to DNA damage Curr Opin Genet Dev 2001 11 71 77 10.1016/S0959-437X(00)00159-3 11163154

[B5] Smith J Tho LM Xu N Gillespie DA The ATM-Chk2 and ATR-Chk1 pathways in DNA damage signaling and cancer Adv Cancer Res 2010 108 73 112 21034966 10.1016/B978-0-12-380888-2.00003-0

[B6] Maya-Mendoza A Petermann E Gillespie DA Caldecott KW Jackson DA Chk1 regulates the density of active replication origins during the vertebrate S phase EMBO J 2007 26 2719 2731 10.1038/sj.emboj.7601714 17491592 PMC1888675

[B7] Petermann E Woodcock M Helleday T Chk1 promotes replication fork progression by controlling replication initiation Proc Natl Acad Sci USA 2010 107 16090 16095 10.1073/pnas.1005031107 20805465 PMC2941317

[B8] Kramer A Mailand N Lukas C Syljuasen RG Wilkinson CJ Nigg EA Bartek J Lukas J Centrosome-associated Chk1 prevents premature activation of cyclin-B-Cdk1 kinase Nat Cell Biol 2004 6 884 891 10.1038/ncb1165 15311285

[B9] Tibelius A Marhold J Zentgraf H Heilig CE Neitzel H Ducommun B Rauch A Ho AD Bartek J Kramer A Microcephalin and pericentrin regulate mitotic entry via centrosome-associated Chk1 J Cell Biol 2009 185 1149 1157 10.1083/jcb.200810159 19546241 PMC2712957

[B10] Gruber R Zhou Z Sukchev M Joerss T Frappart PO Wang ZQ MCPH1 regulates the neuroprogenitor division mode by coupling the centrosomal cycle with mitotic entry through the Chk1-Cdc25 pathway Nat Cell Biol 2011 13 1325 1334 10.1038/ncb2342 21947081

[B11] Tang J Erikson RL Liu X Checkpoint kinase 1 (Chk1) is required for mitotic progression through negative regulation of polo-like kinase 1 (Plk1) Proc Natl Acad Sci USA 2006 103 11964 11969 10.1073/pnas.0604987103 16873548 PMC1567681

[B12] Peddibhotla S Lam MH Gonzalez-Rimbau M Rosen JM The DNA-damage effector checkpoint kinase 1 is essential for chromosome segregation and cytokinesis Proc Natl Acad Sci USA 2009 106 5159 5164 10.1073/pnas.0806671106 19289837 PMC2663996

[B13] Zachos G Black EJ Walker M Scott MT Vagnarelli P Earnshaw WC Gillespie DA Chk1 is required for spindle checkpoint function Dev Cell 2007 12 247 260 10.1016/j.devcel.2007.01.003 17276342 PMC7115955

[B14] Zachos G Gillespie DA Exercising restraints: role of Chk1 in regulating the onset and progression of unperturbed mitosis in vertebrate cells Cell Cycle 2007 6 810 813 10.4161/cc.6.7.4048 17377502

[B15] Stolz A Ertych N Kienitz A Vogel C Schneider V Fritz B Jacob R Dittmar G Weichert W Petersen I Bastians H The CHK2-BRCA1 tumour suppressor pathway ensures chromosomal stability in human somatic cells Nat Cell Biol 2010 12 492 499 10.1038/ncb2051 20364141

[B16] Kellogg DR Moritz M Alberts BM The centrosome and cellular organization Annu Rev Biochem 1994 63 639 674 10.1146/annurev.bi.63.070194.003231 7979251

[B17] Doxsey S McCollum D Theurkauf W Centrosomes in cellular regulation Annu Rev Cell Dev Biol 2005 21 411 434 10.1146/annurev.cellbio.21.122303.120418 16212501

[B18] Doxsey S Zimmerman W Mikule K Centrosome control of the cell cycle Trends Cell Biol 2005 15 303 311 10.1016/j.tcb.2005.04.008 15953548

[B19] Loffler H Lukas J Bartek J Kramer A Structure meets function-centrosomes, genome maintenance and the DNA damage response Exp Cell Res 2006 312 2633 2640 10.1016/j.yexcr.2006.06.008 16854412

[B20] Hinchcliffe EH Miller FJ Cham M Khodjakov A Sluder G Requirement of a centrosomal activity for cell cycle progression through G1 into S phase Science 2001 291 1547 1550 10.1126/science.1056866 11222860

[B21] Khodjakov A Rieder CL Centrosomes enhance the fidelity of cytokinesis in vertebrates and are required for cell cycle progression J Cell Biol 2001 153 237 242 10.1083/jcb.153.1.237 11285289 PMC2185537

[B22] Schmitt E Boutros R Froment C Monsarrat B Ducommun B Dozier C CHK1 phosphorylates CDC25B during the cell cycle in the absence of DNA damage J Cell Sci 2006 119 4269 4275 10.1242/jcs.03200 17003105

[B23] Matsumoto Y Maller JL A centrosomal localization signal in cyclin E required for Cdk2-independent S phase entry Science 2004 306 885 888 10.1126/science.1103544 15514162

[B24] Mikule K Delaval B Kaldis P Jurcyzk A Hergert P Doxsey S Loss of centrosome integrity induces p38-p53-p21-dependent G1-S arrest Nat Cell Biol 2007 9 160 167 10.1038/ncb1529 17330329

[B25] Busch C Barton O Morgenstern E Gotz C Gunther J Noll A Montenarh M The G(2)/M checkpoint phosphatase cdc25C is located within centrosomes Int J Biochem Cell Biol 2007 39 1707 1713 10.1016/j.biocel.2007.04.022 17548228

[B26] Hsu LC White RL BRCA1 is associated with the centrosome during mitosis Proc Natl Acad Sci USA 1998 95 12983 12988 10.1073/pnas.95.22.12983 9789027 PMC23679

[B27] Ciciarello M Mangiacasale R Casenghi M Zaira Limongi M D’Angelo M Soddu S Lavia P Cundari E p53 displacement from centrosomes and p53-mediated G1 arrest following transient inhibition of the mitotic spindle J Biol Chem 2001 276 19205 19213 10.1074/jbc.M009528200 11376010

[B28] Tritarelli A Oricchio E Ciciarello M Mangiacasale R Palena A Lavia P Soddu S Cundari E p53 localization at centrosomes during mitosis and postmitotic checkpoint are ATM-dependent and require serine 15 phosphorylation Mol Biol Cell 2004 15 3751 3757 10.1091/mbc.E03-12-0900 15181149 PMC491834

[B29] Takada S Kelkar A Theurkauf WE Drosophila checkpoint kinase 2 couples centrosome function and spindle assembly to genomic integrity Cell 2003 113 87 99 10.1016/S0092-8674(03)00202-2 12679037

[B30] Tsvetkov L Xu X Li J Stern DF Polo-like kinase 1 and Chk2 interact and co-localize to centrosomes and the midbody J Biol Chem 2003 278 8468 8475 10.1074/jbc.M211202200 12493754

[B31] Hong Y Stambrook PJ Restoration of an absent G1 arrest and protection from apoptosis in embryonic stem cells after ionizing radiation Proc Natl Acad Sci USA 2004 101 14443 14448 10.1073/pnas.0401346101 15452351 PMC521944

[B32] Oricchio E Saladino C Iacovelli S Soddu S Cundari E ATM is activated by default in mitosis, localizes at centrosomes and monitors mitotic spindle integrity Cell Cycle 2006 5 88 92 10.4161/cc.5.1.2269 16319535

[B33] Zhang S Hemmerich P Grosse F Centrosomal localization of DNA damage checkpoint proteins J Cell Biochem 2007 101 451 465 10.1002/jcb.21195 17171639

[B34] Tembe V Henderson BR Protein trafficking in response to DNA damage Cell Signal 2007 19 1113 1120 10.1016/j.cellsig.2007.03.001 17391916

[B35] Cappelli E Townsend S Griffin C Thacker J Homologous recombination proteins are associated with centrosomes and are required for mitotic stability Exp Cell Res 2011 317 1203 1213 10.1016/j.yexcr.2011.01.021 21276791

[B36] De Souza CP Ellem KA Gabrielli BG Centrosomal and cytoplasmic Cdc2/cyclin B1 activation precedes nuclear mitotic events Exp Cell Res 2000 257 11 21 10.1006/excr.2000.4872 10854050

[B37] Jackman M Lindon C Nigg EA Pines J Active cyclin B1-Cdk1 first appears on centrosomes in prophase Nat Cell Biol 2003 5 143 148 10.1038/ncb918 12524548

[B38] Lammer C Wagerer S Saffrich R Mertens D Ansorge W Hoffman I The cdc25B phosphatase is essential for the G2/M phase transition in human cells J Cell Sci 1998 111 2445 2453 9683638 10.1242/jcs.111.16.2445

[B39] Lindqvist A Kallstrom H Lundgren A Barsoum E Rosenthal C Cdc25B cooperates with Cdc25A to induce mitosis but has a unique role in activating cyclin B1-Cdk1 at the centrosome J Cell Biol 2005 171 35 45 10.1083/jcb.200503066 16216921 PMC2171226

[B40] Sibon OC Kelkar A Lemstra W Theurkauf WE DNA-replication/DNA-damage-dependent centrosome inactivation in Drosophila embryos Nat Cell Biol 2000 2 90 95 10.1038/35000041 10655588

[B41] Golan A Pick E Tsvetkov L Nadler Y Kluger H Stern DF Centrosomal Chk2 in DNA damage responses and cell cycle progession Cell Cycle 2010 9 2647 2656 20581449 10.4161/cc.9.13.12121 PMC3233491

[B42] Jallepalli PV Lengauer C Vogelstein B Bunz F The Chk2 tumor suppressor is not required for p53 responses in human cancer cells J Biol Chem 2003 278 20475 20479 10.1074/jbc.M213159200 12654917

[B43] Gillingham AK Munro S The PACT domain, a conserved centrosomal targeting motif in the coiled-coil proteins AKAP450 and pericentrin EMBO Rep 2000 1 524 529 11263498 10.1093/embo-reports/kvd105 PMC1083777

[B44] Petronczki M Lenart P Peters JM Polo on the Rise-from Mitotic Entry to Cytokinesis with Plk1 Dev Cell 2008 14 646 659 10.1016/j.devcel.2008.04.014 18477449

[B45] Archambault V Glover DM Polo-like kinases: conservation and divergence in their functions and regulation Nat Rev Mol Cell Biol 2009 10 265 275 10.1038/nrm2653 19305416

[B46] Steegmaier M Hoffmann M Baum A Lenart P Petronczki M Krssak M Gurtler U Garin-Chesa P Lieb S Quant J BI 2536, a potent and selective inhibitor of polo-like kinase 1, inhibits tumor growth in vivo Curr Biol 2007 17 316 322 17291758 10.1016/j.cub.2006.12.037

[B47] Lenart P Petronczki M Steegmaier M Di Fiore B Lipp JJ Hoffmann M Rettig WJ Kraut N Peters JM The small-molecule inhibitor BI 2536 reveals novel insights into mitotic roles of polo-like kinase 1 Curr Biol 2007 17 304 315 10.1016/j.cub.2006.12.046 17291761

[B48] Mayer TU Kapoor TM Haggarty SJ King RW Schreiber SL Mitchison TJ Small molecule inhibitor of mitotic spindle bipolarity identified in a phenotype-based screen Science 1999 286 971 974 10.1126/science.286.5441.971 10542155

[B49] Holland AJ Cleveland DW Boveri revisited: chromosomal instability, aneuploidy and tumorigenesis Nat Rev Mol Cell Biol 2009 10 478 487 19546858 10.1038/nrm2718 PMC3154738

[B50] Thompson SL Compton DA Examining the link between chromosomal instability and aneuploidy in human cells J Cell Biol 2008 180 665 672 10.1083/jcb.200712029 18283116 PMC2265570

[B51] Lotti LV Ottini L D’Amico C Gradini R Cama A Belleudi F Frati L Torrisi MR Mariani-Costantini R Subcellular localization of the BRCA1 gene product in mitotic cells Genes Chromosomes Cancer 2002 35 193 203 10.1002/gcc.10105 12353262

[B52] Sankaran S Starita LM Groen AC Ko MJ Parvin JD Centrosomal microtubule nucleation activity is inhibited by BRCA1-dependent ubiquitination Mol Cell Biol 2005 25 8656 8668 10.1128/MCB.25.19.8656-8668.2005 16166645 PMC1265743

[B53] Sankaran S Crone DE Palazzo RE Parvin JD Aurora-A kinase regulates breast cancer associated gene 1 inhibition of centrosome-dependent microtubule nucleation Cancer Res 2007 67 11186 11194 10.1158/0008-5472.CAN-07-2578 18056443

[B54] Sankaran S Crone DE Palazzo RE Parvin JD BRCA1 regulates gamma-tubulin binding to centrosomes Cancer Biol Ther 2007 6 1853 1857 10.4161/cbt.6.12.5164 18087219 PMC2643382

[B55] Falck J Mailand N Syljuasen RG Bartek J Lukas J The ATM-Chk2-CDC25A checkpoint pathway guards against radioresistant DNA synthesis Nature 2001 410 842 847 10.1038/35071124 11298456

[B56] Hirao A Kong YY Matsuoka S Wakeham A Ruland J Yoshida H Liu D Elledge SJ Mak TW DNA damage-induced activation of p53 by the checkpoint kinase Chk2 Science 2000 287 1824 1827 10.1126/science.287.5459.1824 10710310

[B57] Lee JS Collins KM Brown AL Lee CH Chung JH hCds1-mediated phosphorylation of BRCA1 regulates the DNA damage response Nature 2000 404 201 204 10.1038/35004614 10724175

[B58] Yang S Kuo C Bisi JE Kim MK PML-dependent apoptosis after DNA damage is regulated by the checkpoint kinase hCds1/Chk2 Nat Cell Biol 2002 4 865 870 10.1038/ncb869 12402044

[B59] Stevens C Smith L La Thangue NB Chk2 activates E2F-1 in response to DNA damage Nat Cell Biol 2003 5 401 409 10.1038/ncb974 12717439

[B60] Tan Y Raychaudhuri P Costa RH Chk2 mediates stabilization of the FoxM1 transcription factor to stimulate expression of DNA repair genes Mol Cell Biol 2007 27 1007 1016 10.1128/MCB.01068-06 17101782 PMC1800696

[B61] Enomoto M Goto H Tomono Y Kasahara K Tsujimura K Kiyono T Inagaki M Novel positive feedback loop between Cdk1 and Chk1 in the nucleus during G2/M transition J Biol Chem 2009 284 34223 34230 10.1074/jbc.C109.051540 19837665 PMC2797192

[B62] Matsuyama M Goto H Kasahara K Kawakami Y Nakanishi M Kiyono T Goshima N Inagaki M Nuclear Chk1 prevents premature mitotic entry J Cell Sci 2011 124 2113 2119 10.1242/jcs.086488 21628425

[B63] Carrassa L Sanchez Y Erba E Damia G U2OS cells lacking Chk1 undergo aberrant mitosis and fail to activate the spindle checkpoint J Cell Mol Med 2009 13 1565 1576 19778378 10.1111/j.1582-4934.2008.00362.x PMC3828867

[B64] Yang C Tang X Guo X Niikura Y Kitagawa K Cui K Wong ST Fu L Xu B Aurora-B mediated ATM serine 1403 phosphorylation is required for mitotic ATM activation and the spindle checkpoint Mol Cell 2011 44 597 608 10.1016/j.molcel.2011.09.016 22099307 PMC3228519

[B65] Lee KJ Lin YF Chou HY Yajima H Fattah KR Lee SC Chen BP Involvement of DNA-dependent protein kinase in normal cell cycle progression through mitosis J Biol Chem 2011 286 12796 12802 10.1074/jbc.M110.212969 21330363 PMC3069479

[B66] Townsend K Mason H Blackford AN Miller ES Chapman JR Sedgwick GG Barone G Turnell AS Stewart GS Mediator of DNA damage checkpoint 1 (MDC1) regulates mitotic progression J Biol Chem 2009 284 33939 33948 10.1074/jbc.M109.009191 19826003 PMC2797164

[B67] Campeau E Ruhl VE Rodier F Smith CL Rahmberg BL Fuss JO Campisi J Yaswen P Cooper PK Kaufman PD A versatile viral system for expression and depletion of proteins in mammalian cells PLoS One 2009 4 e6529 10.1371/journal.pone.0006529 19657394 PMC2717805

[B68] Rodier F Coppe JP Patil CK Hoeijmakers WA Munoz DP Raza SR Freund A Campeau E Davalos AR Campisi J Persistent DNA damage signalling triggers senescence-associated inflammatory cytokine secretion Nat Cell Biol 2009 11 973 979 10.1038/ncb1909 19597488 PMC2743561

[B69] Hansen DV Loktev AV Ban KH Jackson PK Plk1 regulates activation of the anaphase promoting complex by phosphorylating and triggering SCFbetaTrCP-dependent destruction of the APC Inhibitor Emi1 Mol Biol Cell 2004 15 5623 5634 10.1091/mbc.E04-07-0598 15469984 PMC532041

[B70] Wang J Beauchemin M Bertrand R Phospho-Bcl-xL(Ser62) plays a key role at DNA damage-induced G 2 checkpoint Cell Cycle 2012 11 2159 2169 10.4161/cc.20672 22617334 PMC3368867

