# Supplementary figures and images for: Cell cycle-dependent localization of CHK2 at centrosomes during mitosis
Source: Cell Div. 2013 May 16;8:7. doi: 10.1186/1747-1028-8-7 (PMC3668180; doi:10.1186/1747-1028-8-7)

Additional file 1

A

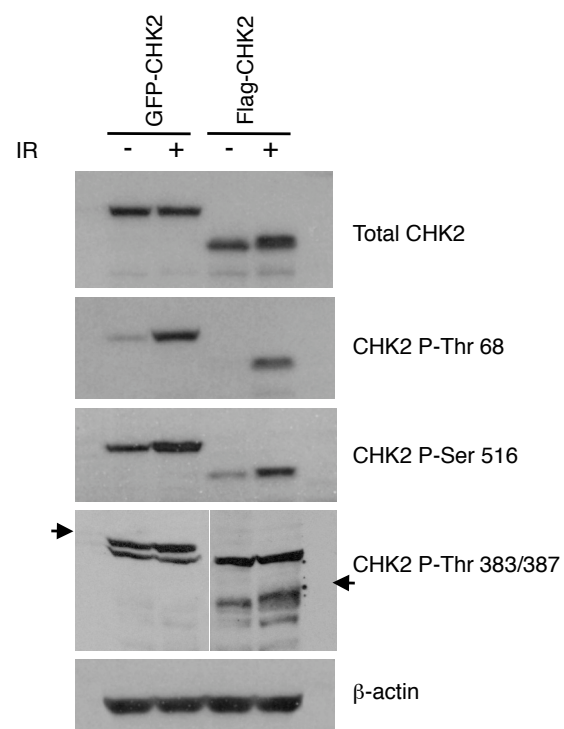

B

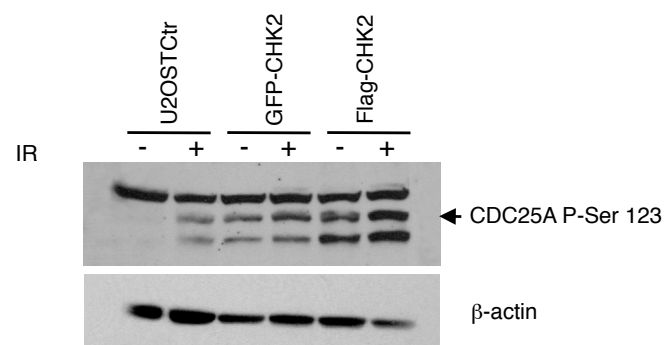

Supplement: Additional file 1 — The exogenous GFP-CHK2 and Flag-CHK2 fusion proteins are functional kinases. U2OS GFP-CHK2 and U2OS Flag-CHK2 cell lines were incubated with doxycycline for 48 h to induce transgene expression and cells were exposed to γ-IR (10 Gy ). (A) 1 h after genotoxic insult whole-cell lysates were prepared and phosphorylation of CHK2 fusion proteins on Thr 68, Thr 383/387 and Ser 516 was assessed by Western blotting using the indicated antibodies. The arrows indicate GFP-CHK2 and Flag-CHK2 proteins phoshorylated on Thr383/387. (B) The phosphorylation of the CHK2 substrate CDC25 A (Ser123) was analyzed by Western blotting of protein extracts prepared 8 h after irradiation. The arrow indicates the band corresponding to P-Ser123-CDC25A. Both GFP-CHK2 and Flag-CHK2 retain full kinase activity. [file 1747-1028-8-7-S1.pdf]

Additional file 2

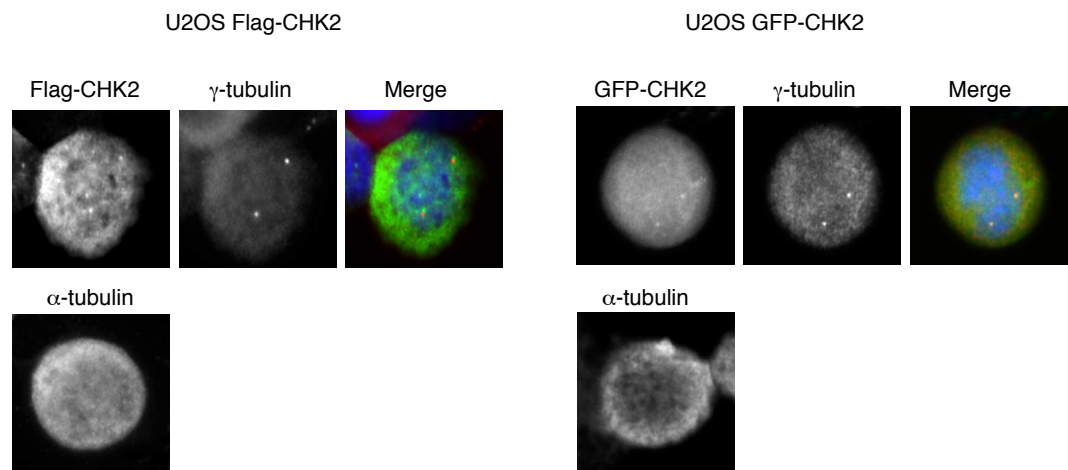

Supplement: Additional file 2 — During mitosis the localization of CHK2 at the centrosomes is microtubules independent. Doxycycline-induced U2OS GFP-CHK2 and U2OS Flag-CHK2 cell lines were incubated for 16 h in nocodazole (0.3 μM) to arrest cells in prometaphase. Cells were treated for an additional hour with 10 μM nocodazole prior to be fixed and stained with anti-γ-tubulin antibody (red) to stain the centrosomes. GFP-CHK2 was visualized by direct fluorescence and Flag-CHK2 was immunostained with an anti-Flag antibody (green). To control microtubules depolymerization cells were also stained for α-tubulin. [file 1747-1028-8-7-S2.pdf]

# Additional file 3

**A**

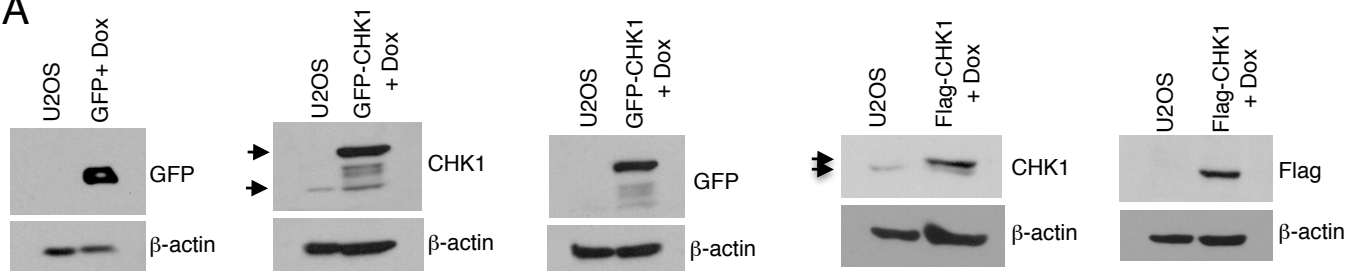

**B**

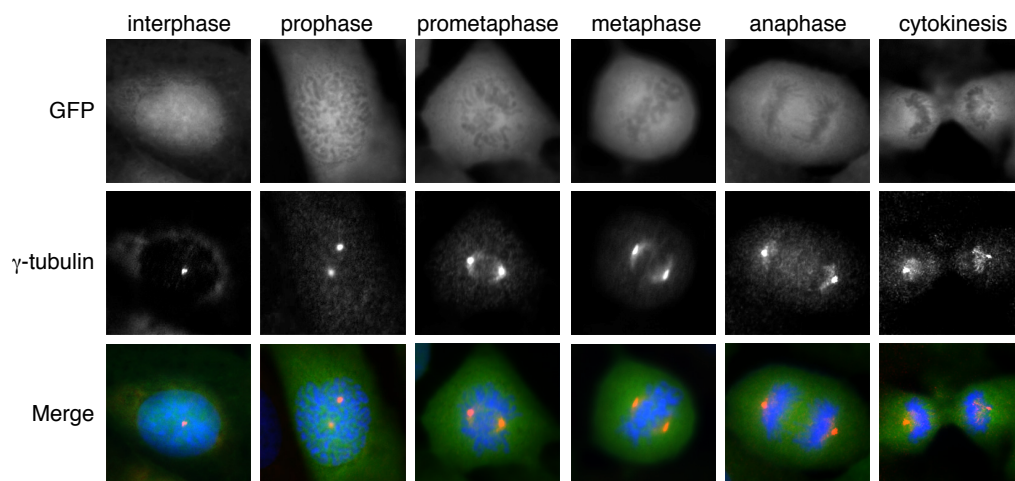

**C**

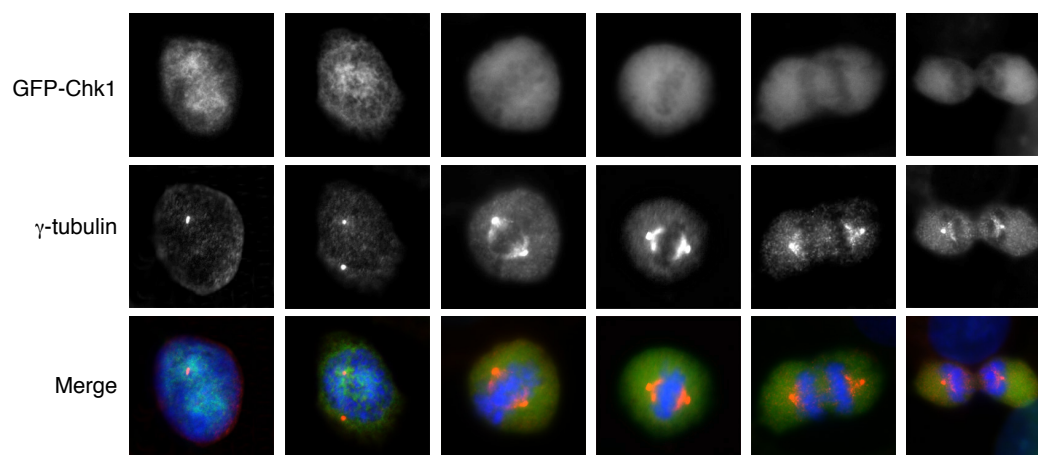

**D**

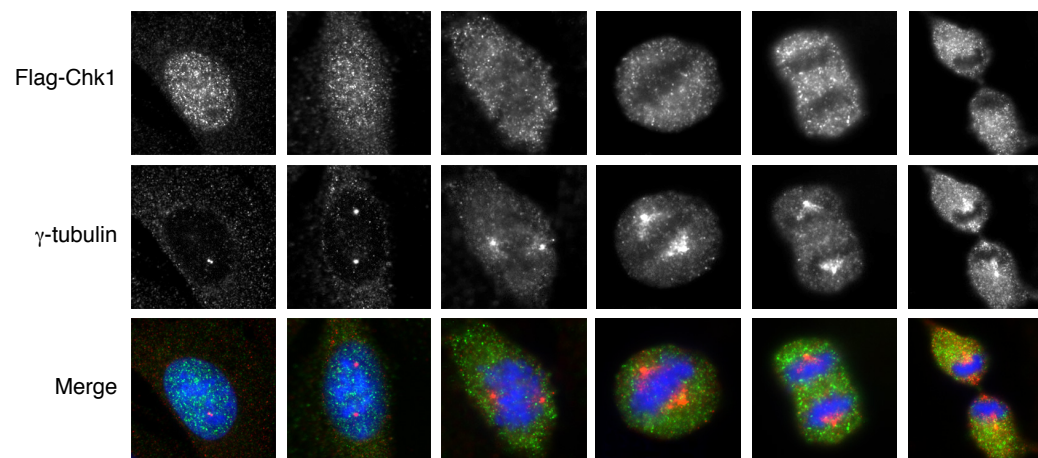

Supplement: Additional file 3 — GFP, GFP-CHK1 and Flag-CHK1 do not localize to the centrosomes. U2OS stably transduced with lentiviruses coding for GFP, GFP-CHK1 or Flag-CHK1 were exposed to doxycycline at 5 ng/ml, 10 ng/ml and 20 ng/ml. (A) 48 h following doxycycline addition cells were collected. The expression of exogenous proteins was analyzed by Western blotting using the indicated antibodies. The arrows denote endogenous and exogenous CHK1 proteins. β-actin was used as loading control. (B-D) 48 h post-induction, cells were fixed and immunostained with anti-γ-tubulin antibody (red) and costained with DAPI (blue). The localization of GFP and GFP-CHK1 was observed by direct fluorescence and Flag-CHK1 was immunostained with an anti-Flag antibody (green). Cells in interphase and various phases of mitosis were selected. [file 1747-1028-8-7-S3.pdf]

A

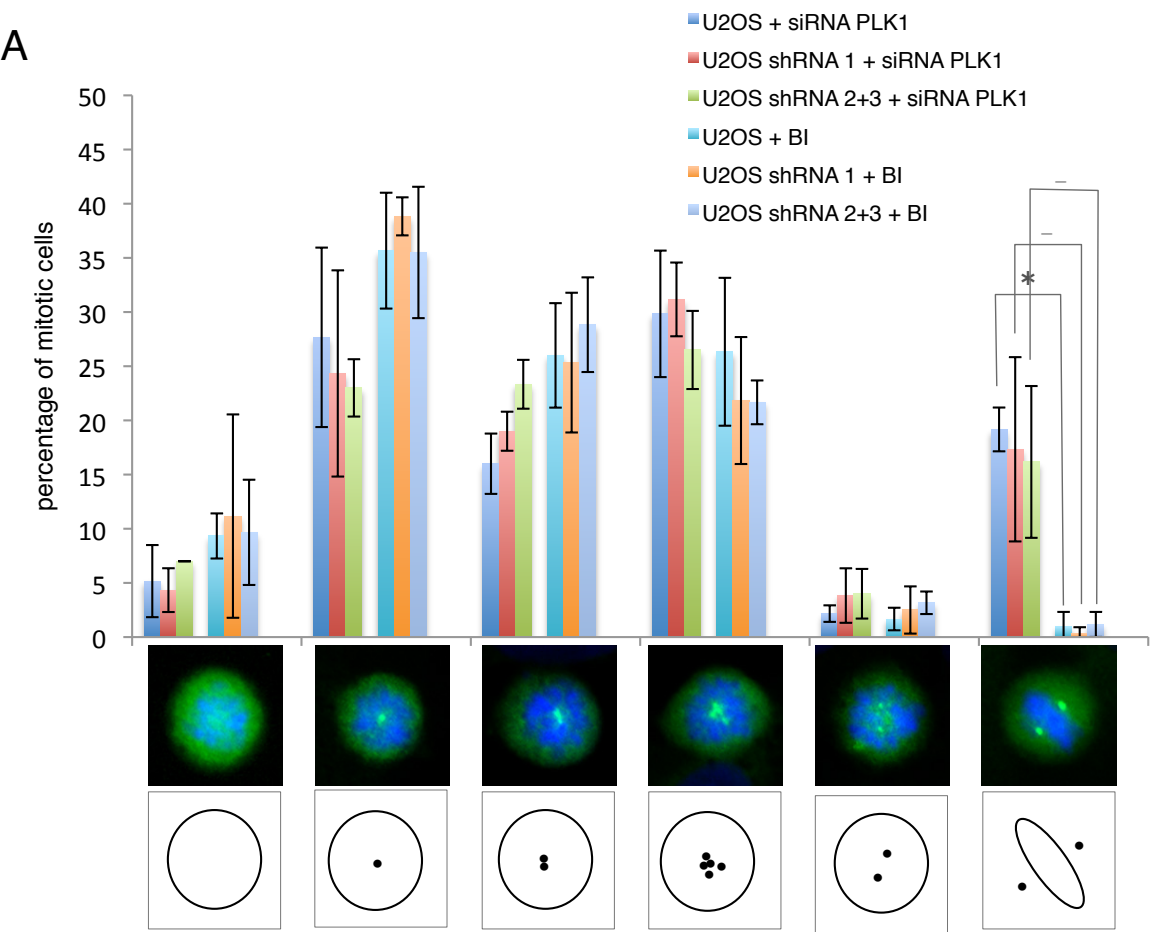

B

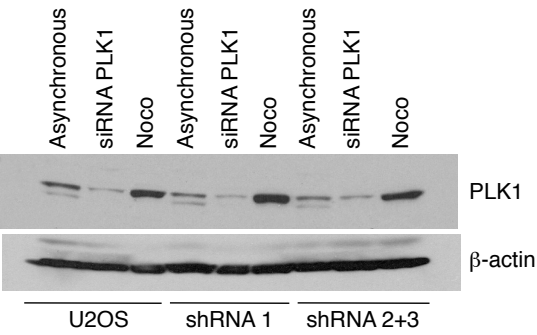

Supplement: Additional file 6 — Quantification of centrosome separation in mitotic cells. (A) Control U2OS cells or cells stably transduced with CHK2 shRNA 1 or CHK2 shRNA 2 + 3 were transfected with a siRNA directed against PLK1 or incubated with BI 2536 (100 nM). 24 h following transfection or 16 h after treatment with BI 2536, cells were fixed and stained with anti-γ-tubulin antibody and DAPI. Representative images of the mitotic-arrested cells are shown. The percentage of each mitotic cellular population was measured. Error bars represent the mean ± s.d. of 3 independent experiments, each experiment monitoring 200 mitotic cells (*P < 0.05; _ P > 0,05). (B) Western blot analysis of PLK1 expression. Cell lysates from PLK1 siRNA-transfected U2OS cells were prepared from mitotic cells collected by shake-off 24 h post-transfection. Protein extracts prepared from asynchronous cells or mitotic cells collected by shake-off 24 h following nocodazole treatment serves as control. [file 1747-1028-8-7-S6.pdf]

Additional file 7

A

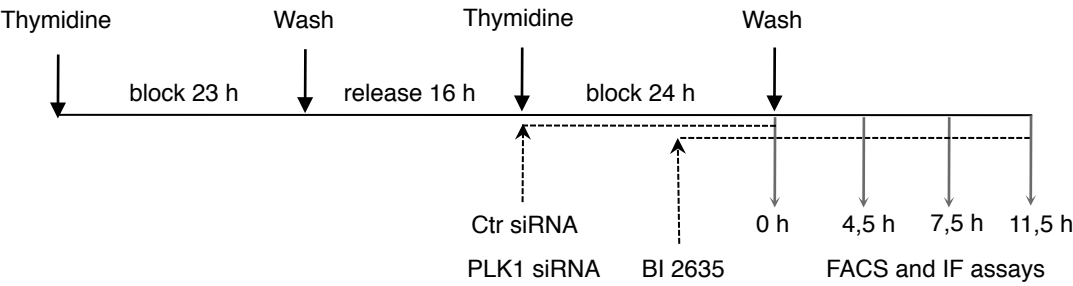

B

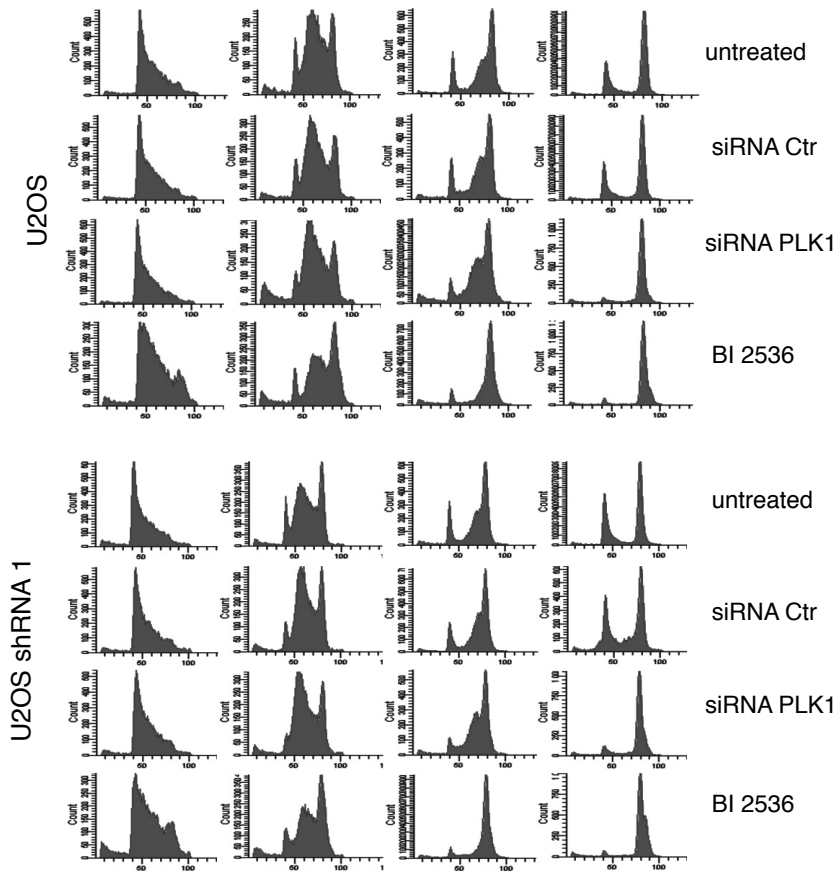

C

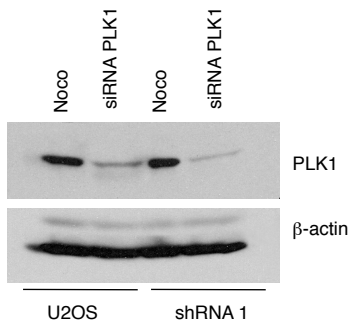

D

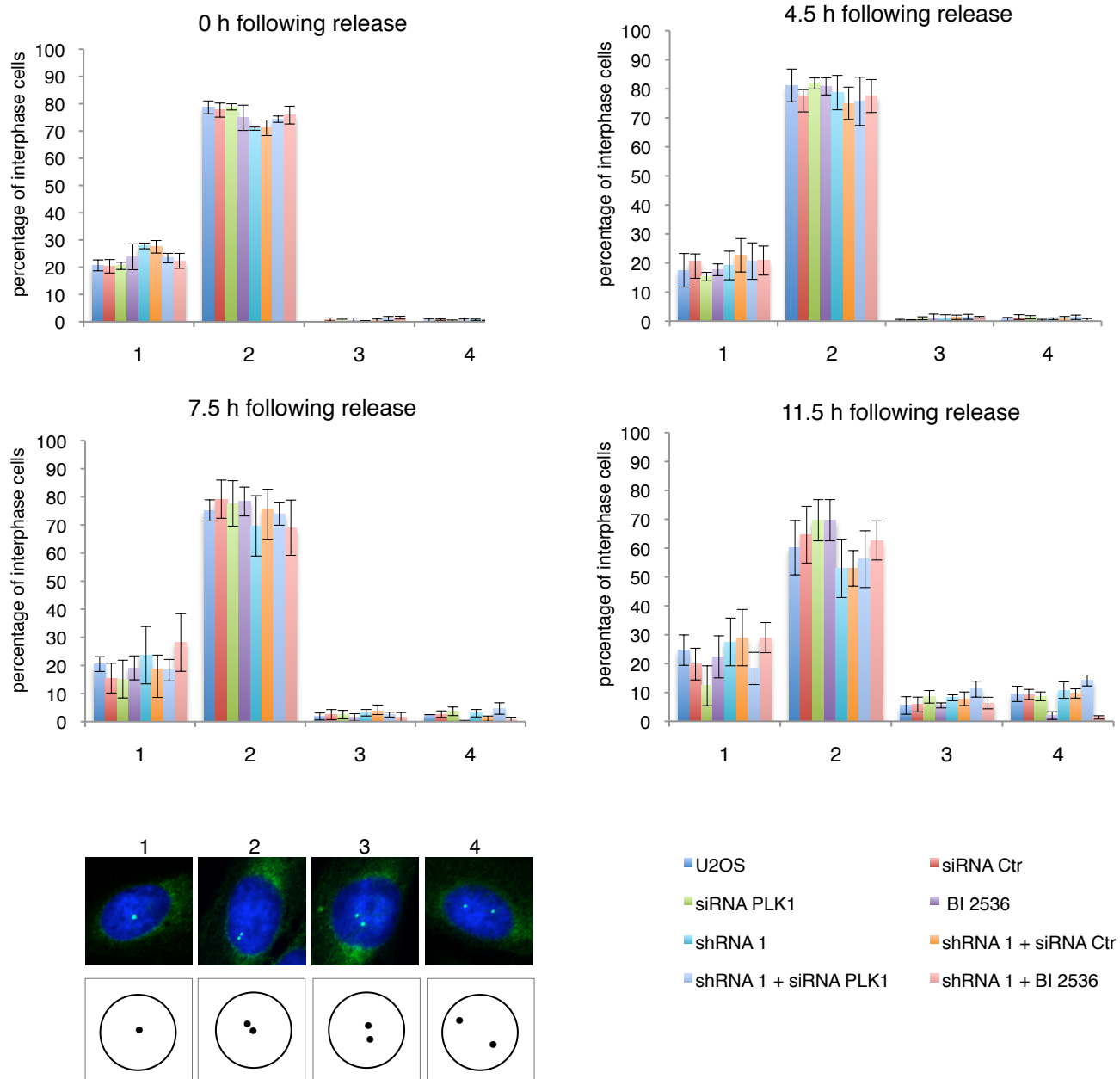

Supplement: Additional file 7 — Quantification of centrosomes duplication/separation in interphase. (A) Experimental procedure. Control U2OS cells or cells stably transduced with CHK2 shRNA 1 were synchronized at the G1/S boundary by a double thymidine block (DTB). At the indicated times during the cell cycle synchronization protocol, cells were transfected with control or PLK1 siRNAs, incubated with BI 2536 or left untreated. (B) After release from second thymidine block, cell synchronization was confirmed by FACS analysis at the indicated times. (C) The inhibition of PLK1 expression was confirmed by Western blotting. Cell lysates from PLK1 siRNA-transfected cells were prepared from mitotic cells collected by shake-off 11,5 h after release from DTB. Protein extracts prepared from mitotic cells collected 24 h following nocodazole treatment serves as control. (D) At each time point after release, cells were fixed and stained with anti-γ-tubulin antibody and DAPI. The interphase cells with one or two unseparated/separated centrosomes were divided in 4 patterns, as shown in representative images, and cells in each pattern were quantified. Error bars represent the mean ± s.d. of 3 independent experiments, each experiment monitoring 200 interphase cells. [file 1747-1028-8-7-S7.pdf]
